# Longitudinal Radiological Findings in Patients With COVID-19 With Different Severities: From Onset to Long-Term Follow-Up After Discharge

**DOI:** 10.3389/fmed.2021.711435

**Published:** 2021-09-21

**Authors:** Yajing Zhao, Dongdong Wang, Nan Mei, Bo Yin, Xuanxuan Li, Yingyan Zheng, Anling Xiao, Xiangrong Yu, Xiaohui Qiu, Yiping Lu, Li Liu

**Affiliations:** ^1^Department of Radiology, Huashan Hospital, Fudan University, Shanghai, China; ^2^Department of Radiology, Fuyang Second People's Hospital, Fuyang, China; ^3^Department of Radiology, Zhuhai People's Hospital, Zhuhai, China; ^4^Department of Radiology, Bozhou People's Hospital, Bozhou, China; ^5^Department of Radiology, Shanghai Cancer Center, Fudan University, Shanghai, China

**Keywords:** COVID-19, longitudinal, follow-up, prognosis, parenchymal bands

## Abstract

**Objective:** This study aimed to investigate the evolution of radiological findings in the patients with coronavirus disease 2019 (COVID-19) pneumonia with different severities from onset to 1-year follow-up and identify the predictive factors for different pulmonary lesion absorption status in the patients infected with COVID-19.

**Methods:** A retrospective study was performed on the clinical and radiological features of 175 patients with COVID-19 pneumonia hospitalized at three institutions from January 21 to March 20, 2020. All the chest CT scans during hospitalization and follow-ups after discharge were collected. The clinical and radiological features from the chest CT scans both at the peak stage and before discharge from the hospital were used to predict whether the pulmonary lesions would be fully absorbed after discharge by Cox regression. Then, these patients were stratified into two groups with different risks of pulmonary lesion absorption, and an optimal timepoint for the first CT follow-up was selected accordingly.

**Results:** A total of 132 (75.4%) patients were classified into the non-severe group, and 43 (24.6%) patients were classified into the severe group, according to the WHO guidelines. The opacification in both the groups changed from ground-glass opacity (GGO) to consolidation and then from consolidation to GGO. Among the 175 participants, 135 (112 non-severe and 23 severe patients with COVID-19) underwent follow-up CT scans after discharge. Pulmonary residuals could be observed in nearly half of the patients (67/135) with the presentation of opacities and parenchymal bands. The parenchymal bands in nine discharged patients got fully absorbed during the follow-up periods. The age of patient [hazard ratio (*HR*) = 0.95, 95% *CI*, 0.95–0.99], level of lactate dehydrogenase (LDH) (*HR* = 0.99; 95% *CI*, 0.99–1.00), level of procalcitonin (*HR* = 8.72; 95% *CI*, 1.04–73.03), existence of diffuse lesions (*HR* = 0.28; 95% *CI*, 0.09–0.92), subpleural distribution of lesions (*HR* = 2.15; 95% CI, 1.17–3.92), morphology of residuals (linear lesion: *HR* = 4.58, 95% *CI*, 1.22–17.11; nodular lesion: *HR* = 33.07, 95% *CI*, 3.58–305.74), and pleural traction (*HR* = 0.41; 95% *CI*, 0.22–0.78) from the last scan before discharge were independent factors to predict the absorption status of COVID-19-related pulmonary abnormalities after discharge. According to a Kaplan–Meier analysis, the probability of patients of the low-risk group to have pulmonary lesions fully absorbed within 90 days reached 91.7%.

**Conclusion:** The development of COVID-19 lesions followed the trend from GGO to consolidation and then from consolidation to GGO. The CT manifestations and clinical and laboratory variables before discharge could help predict the absorption status of pulmonary lesions after discharge. The parenchymal bands could be fully absorbed in some COVID-19 cases. In this study, a Cox regression analysis indicated that a timepoint of 3 months since onset was optimal for the radiological follow-up of discharged patients.

## Introduction

The contemporary emerging coronavirus disease 2019 (COVID-19) has become an international public health event, and the death toll reached over 3 million as of April 20, 2021 (1, 2). Severe acute respiratory syndrome coronavirus-2 (SARS-CoV-2) is identified as the pathogen of COVID-19, which shares a structural similarity with severe acute respiratory syndrome coronavirus (SARS-CoV), binding with angiotensin-converting enzyme 2 (ACE2) receptors *via* the spike protein (S-protein) to invade host cells (3, 4). Although multiple organs were reported to be affected by SARS-CoV-2, given the high expression of ACE-2 receptor in type II lung cells, the respiratory system was still the first and main organ to be damaged (5, 6).

Currently, chest CT is the main imaging facility to detect and manage COVID-19 related pneumonia (7, 8). Based on high accuracy in COVID-19 diagnosis, chest CT imaging was once placed in an unusually important position, bearing the potential to replace nucleic acid amplification test (NAAT) at the beginning of the outbreak. With the improvement of the accuracy and speed of the NAAT, chest CT imaging is no longer a determinant diagnostic tool for COVID-19 but remains an important tool for disease evaluation. Multiple studies have described the short-term CT manifestations in the patients infected with COVID-19, while few studies have investigated their long-term CT findings after discharge (9–11). According to the updated guidelines of the Government of China, patients with COVID-19 were required to undergo follow-ups at 2 and 4 weeks after discharge with or without imaging surveillance (12). In the guidelines published by the British Thoracic Society, imaging follow-up is recommended for all the patients with COVID-19 admitted to the hospital 3 months after discharge based on the experience of SARS and Middle East respiratory syndrome (MERS) (6, 13, 14). Without enough follow-up studies, the optimal time for follow-up imaging to assess radiological clearance in COVID-19 is unclear. Therefore, it is important to clarify the dynamic changes in COVID-19 related pulmonary abnormalities from onset to the convalescence stage that could help us understand the disease comprehensively and make customized follow-up plans for different individuals.

It has been over 1 year since the outbreak of COVID-19 in China. Most patients with COVID-19 were discharged and had already undergone several follow-up CT scans. In this study, we collected all CT scans from the survivors of COVID-19 in multiple institutions to investigate the longitudinal radiological development in patients with different clinical types during hospitalization and follow-ups, aiming to depict the longitudinal changes in pulmonary lesions, select the important features to impact the resolution of chest CT abnormalities in patients with COVID-19, and provide evidence to make optimal imaging surveillance plans for different patients.

## Materials and Methods

### Patient Cohort

The institutional review board approved this multicenter retrospective study and waived the requirement of written informed consent. Deidentified data were used to prevent any leak of the patient‘s privacy. The patients admitted to the three hospitals in Anhui and Hubei Provinces in China from January 21 to March 20, 2020 and who met the following requirements were enrolled in our study: (1) patients with laboratory-confirmed COVID-19 infection by NAAT; (2) patients who had pneumonia manifestations on at least one chest CT scan during hospitalization; (3) at least two chest CT scans during hospitalization (at admission and before discharge) were required; and (4) no significant artifacts were presented in the CT images. The exclusion criteria were as follows: (a) patients who were transferred from other hospitals (*n* = 5); (b) patients whose chest CT findings were negative (*n* = 11); (c) lack of chest CT scans at the time of admission (*n* = 28); and (d) the unqualified CT scans or images (*n* = 2).

**Figure 1:**
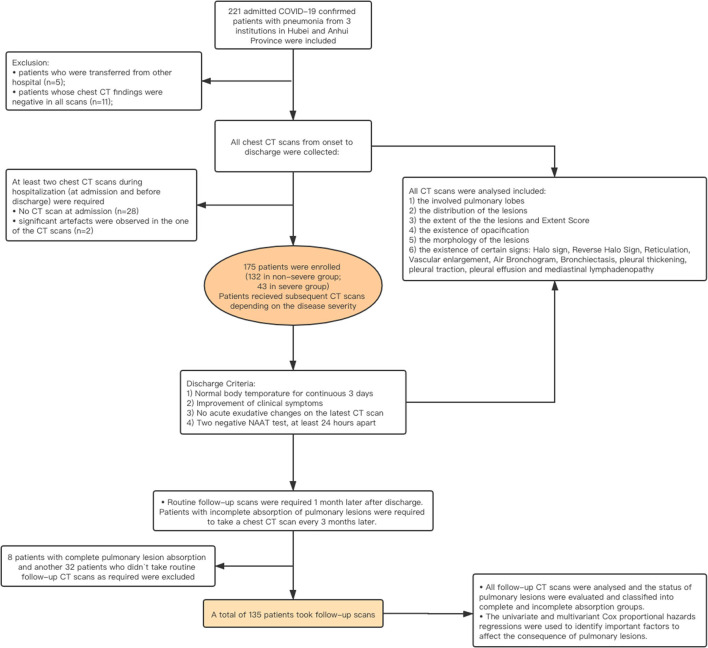
Workflow of the study.

The epidemiological history, main complaints, underlying comorbidity, chest CT imaging features, and laboratory test results at the time of admission were recorded. The clinical types were initially determined based on the guidelines proposed by China and WHO, such as mild, moderate, severe, and critical types (12, 15). The criteria are provided in Supplementary Table 1. The mild and moderate cases were categorized into the **non-severe group**, while severe and critical types were classified into the **severe group**. All the enrolled patients with COVID-19 underwent CT scans at admission and before discharge. According to the guidelines version 5.0–7.0 issued by the Government of China, the treatment used during hospitalization was also recorded. Referring to the guidelines version 7 of China, all the patients were recommended to undergo at least one follow-up CT scan within 1 month after discharge (16). If abnormalities were detected, the patient was recommended to undergo a follow-up CT 3 months later. The workflow of the study is shown in Figure 1.

### Clinical Course Assessment

The onset date was recorded as the day when symptoms first appeared. The onset date, admission date, date of transfer to the intensive care unit (ICU), date of transfer out of the ICU, two NAAT test dates, discharge date, and each CT scan date, were recorded. The duration of admission was calculated from the 1st day of hospitalization to the day of discharge. The clinical course was calculated from the day of onset to the day of discharge. The absorption period was calculated from the day of onset to the day of a CT scan with normal appearance.

### CT Protocol

A total of 175 patients were enrolled from three institutions in Anhui and Hubei Provinces, China. Unenhanced CT scans were applied to all the patients. Patients were imaged with a 64-section CT scanner (Aquilion 64, Toshiba Medical Systems, Otawara, Japan/Somantom Sensation, Siemens, Germany) or a 16-section CT scanner (Emotion 16, Siemens Medical Solutions, Erlangen, Germany). The parameters used for the scanning protocol were as followed: patient in the supine position; end inspiratory acquisition; tube voltage, 100–120 kV; tube current–exposure time product, 200–300 mAs; pitch, 1.375 and 0.9125; and section thickness after reconstruction, 1.25 mm. The images were displayed at lung (window width, 1,500 HU; window level, −500 HU) and mediastinal (window width, 320 HU; window level, 40 HU) windows.

### CT Manifestation Analysis

All the imaging data were analyzed with consensus by two experienced radiologists (10 and 8 years of clinical experience) who were blinded to the clinical data and consensus was reached after negotiation.

For all the CT scans, 15 pulmonary lesion related parameters were collected as listed below: (a) the involved pulmonary lobes; (b) the location of pulmonary lesions, such as subpleural, central, or both; (c) the extent of the lesions, such as unifocal, multifocal, and diffuse; (d) a semi-quantitative scoring system (Extent Score) was used to estimate the extent (17, 18). Each lung was divided into upper (above the tracheal carina), lower (below the inferior pulmonary vein), and middle (in between) zones, and each zone was scored based on the following criteria: 0, 0%; 1, < 25%; 2, 25–49%; 3, 50–74%; and 4, > 75%. The extent of abnormalities was determined by the summation of scores (possible range 0–24); (e) the existence of opacification, such as ground-glass opacity (GGO), mixed (mainly GGO, mainly consolidation), and consolidation; (f) the morphology of the lesions, such as nodular (characterized on CT scans as a rounded or irregular opacity, well or poorly defined, measuring up to 3 cm in diameter), linear (fine linear opacity), patchy (isolated focal lesions with no nodular/linear shape in the segment), and large patchy (large fused lesions involving multiple segments); the existence of a/an (g) “crazy-paving” pattern; (h) halo sign; (i) reversed halo sign (RHS); (j) reticular pattern; (k) vascular enlargement; (l) air bronchogram; (m) bronchiolectasis; (n) pleural thickening; (o) pleural traction; (p) of pleural effusion; and (q) mediastinal lymphadenopathy (the minimal axial diameter >1 cm) (19). The descriptions of radiological features used the definitions compiled by the Fleischner Society (20).

For follow-up scans, except for the assessments above, each CT scan was labeled normal or abnormal according to the existence of pulmonary lesions for further analysis.

### Statistical Analysis

All the statistical analyses were performed with R software (version 3.5.3; R Foundation for Statistical Computing, Vienna, Austria). The Shapiro–Wilk test was used to evaluate the distribution type, and Bartlett's test was used to evaluate the homogeneity of variance. Normally distributed data are expressed as the mean ± SD. Non-normally distributed data and ordinal data are expressed as medians [interquartile ranges (IQRs)]. The categorical variables were summarized as counts and percentages. Comparisons of non-paired quantitative data were evaluated using the Mann–Whitney *U*-test and Wilcoxon's test. Comparisons of categorized data were evaluated by the chi-square test and Fisher's test. A value of *p* < 0.05 was defined as statistically significant.

To identify the important variables to stratify patients with/without pulmonary residual lesions, all the clinical and laboratory variables and CT manifestations before discharge and at the peak stage were evaluated by univariate Cox proportional hazards regression analyses and factors with a value of *p* < 0.05 were further assessed in the multivariate Cox proportional hazards regression analysis.

## Results

### Clinical Characteristics

A total of 175 patients (102 men and 73 women) were included in this study. The average age was 44.75 ± 13.65 years old. A total of 132 patients (75.4%, all in moderate type) were classified into the **non-severe** group, and 43 patients (24.6%, 41 in severe type and 2 in critical type) were classified into the **severe** group. There were no patients with **mild** type recruited in this study. Patients in the **severe** group were older than those in the **non-severe** group (50.63 ± 13.29 vs. 42.89 ± 13.27 years old, respectively, *p* = 0.001) and were hospitalized for 23.5 (20–30) days, longer than the hospitalization duration of **non-severe** patients [18 (15–23) days, *p* < 0.001]. Severe patients were more likely to have severe symptoms at onset. Chest tightness was reported in 25.6% of the severe group and 9.9% of the non-severe group. Underlying comorbidities, especially cardiovascular and endocrinal diseases were more frequently found in the severe patients. Lower lymphocyte cell counts, lower lactate dehydrogenase (LDH), and higher aminotransferase (AST) levels were seen in severe cases (Tables 1, 2).

**Table 1:** Predominant clinical characteristics and laboratory findings in patients from non-severe and severe group.

**Characteristics**	**All patients (***n*** = 175)**	**Non-severe group (***n*** = 132)**	**Severe group (***n*** = 43)**	***p*** **-value**
**Age**	44.79 ± 13.65	42.89 ± 13.27	50.63 ± 13.29	**0.001**
**Gender, male/female**	102/73	73/59	29/14	0.221
**Course**				
Admission duration^**^, median (1/4–3/4 quantile), day	19 (16–26)	18.00 (15–23)	23.50 (20–30)	**<0.001**
ICU duration, median (1/4–3/4 quantile), day	9.50 (6–15)	/	9.50 (6–15)	/
Course^***^, median (1/4–3/4 quantile), day	25 (20–31)	24 (19–29)	31 (27–35)	**<0.001**
**Symptom**				
Fever	148 (84.6%)	111 (84.1%)	37 (86.1%)	0.948
Cough	94 (53.7%)	68 (51.5%)	26 (60.5%)	0.340
Chest tightness	24 (13.7%)	13 (9.8%)	11 (25.6%)	**0.019**
Fatigue	25 (14.3%)	17 (12.9%)	8 (18.6%)	0.496
Diarrhea	5 (2.9%)	5 (3.8%)	0 (0%)	0.336^*^
Headache	5 (2.9%)	5 (3.8%)	0 (0%)	0.336^*^
Others	1 (2.0%)	1 (0.8%)	0 (0%)	1.000^*^
**Underlying comorbidity**				
**Yes/no**	48/127	30/102	18/25	**0.025**
Endocrine system disease	23 (13.1%)	13 (9.9%)	10 (23.3%)	**0.045**
Digestive system disease	10 (5.7%)	8 (6.1%)	2 (4.7%)	1.000
Cardiovascular and cerebrovascular disease	11 (6.3%)	5 (3.8%)	6 (14.0%)	0.028^*^
Malignancy	3 (1.7%)	1 (0.8%)	2 (4.7%)	0.150^*^
Mental disease	2 (1.1%)	1 (0.8%)	1 (2.3%)	0.432^*^
Respiratory system disease	2 (1.1%)	1 (0.8%)	1 (2.3%)	0.432^*^
Other	5 (2.9%)	4 (3.0%)	1 (2.3%)	1.000^*^
**Laboratory test**				
White blood cell count, mean ± sd, × 10^9^/L	5.53 ± 2.33	5.32 ± 1.96	6.35 ± 3.29	0.274
Lymphocyte count, mean ± sd, × 10^9^/L	1.09 ± 0.44	1.19 ± 0.43	0.80 ± 0.35	**<0.001**
Lactate dehydrogenase, mean ± sd, U/L	260.43 ± 95.46	242.80 ± 65.69	341.39 ± 154.50	<0.001^*^
C-reactive protein, mean ± sd, mg/L	28.37 ± 35.67	21.18 ± 26.76	50.28 ± 48.70	<0.001^*^
Procalcitonin, median (range), ng/mL	0.09 ± 0.38	0.10 ± 0.42	0.06 ± 0.05	0.339^*^
Alanine aminotransferase, mean ± sd, U/L	39.50 ± 44.99	38.60 ± 47.59	42.55 ± 35.20	0.078^*^
Aspartate aminotransferase, mean ± sd, U/L	32.76 ± 30.07	31.99 ± 32.95	35.30 ± 17.52	0.008^*^
**Therapeutic strategy**				
Antiviral therapy	169 (96.6%)	127 (96.2%)	42 (97.7%)	1.000^*^
Oxygen inhalation	121 (69.1%)	78 (59.1%)	43 (100.0%)	<0.001^*^
Antibiotic treatment	81 (46.3%)	51 (38.6%)	30 (69.8%)	<**0.001**
Interferon therapy	44 (25.1%)	37 (28.0%)	7 (16.3%)	0.182
Glucocorticoid therapy	7 (4.0%)	4 (3.0%)	3 (7.0%)	**0.001**

* *Wilcoxon's rank-sum test and Fisher's exact test were used if non-normal distribution or heterogenous variance of the data was detected*.

** *Admission duration was calculated from the 1st day of hospitalization to the day of discharge*.

*** *Course was calculated from the day of onset to the day of discharge*.

*The p value in bold indicates that P is less than 0.05 and is statistically significant*.

**Table 2:** Chest CT imaging manifestations in non-severe and severe groups from onset to discharge.

**Imaging manifestation**	**All scans (714 scans from 175 patients)**	**Non-severe group scans (509 scans from 135 patients)**	**Severe group scans (205 scans from 43 patients)**	***p*** **-value**
Number of Scans per case	4.07 ± 1.54	3.86 ± 1.45	4.72 ± 1.67	**0.004**
**Period**				
Between Onset and the CT Scan, median (1/4–3/4 quantile), day	14 (9–20)	14 (8–19)	16 (10–23)	0.003^*^
Between Onset and the 1st CT Scan, median (1/4–3/4 quantile), day^**^	7 (5–10)	7 (4–10)	7 (4–11.5)	0.581
Between Onset and the last CT Scan, median (1/4-3/4 quantile), day^**^	22 (16.5–26)	20 (16–25)	25 (20.5–29)	**0.004**
**Involved lobes**				
Right upper lobe	535 (74.9%)	339 (66.6%)	196 (95.6%)	**<0.001**
Right middle lobe	427 (59.8%)	266 (52.3%)	161 (78.5%)	**<0.001**
Right lower lobe	608 (85.2%)	412 (80.9%)	196(95.6%)	**<0.001**
Center upper lobe	511 (71.6%)	326 (64.1%)	185 (90.2%)	**<0.001**
Right lower lobe	632 (88.5%)	431 (84.7%)	201 (98.1%)	**<0.001**
**Location of Lesions**				
Subpleural	410 (57.4%)	325 (63.9%)	85 (41.5%)	<0.001^*^
Central	7 (1.0%)	5 (1.0%)	2 (1.0%)	
Both	282 (39.5%)	165 (32.4%)	117 (57.1%)	
None	15 (2.1%)	14 (2.8%)	1 (0.5%)	
**Extent of lesions**				
Unifocal	37 (5.2%)	36 (7.1%)	1 (0.5%)	<0.001^*^
Multi-focal	443 (62.0%)	367 (72.1%)	76 (37.1%)	
Diffuse	219 (60.7%)	92 (18.1%)	127 (62.0%)	
None	15 (2.1%)	14 (2.8%)	1 (0.5%)	
Extent Score	7.19 ± 5.13	5.19 ± 3.54	11.16 ± 6.20	<0.001^*^
**The existence of opacification**				
GGO	139 (19.5%)	119 (23.4%)	20 (9.8%)	<0.001^*^
Mixed (mainly GGO)	297 (41.6%)	208 (40.9%)	89 (43.4%)	
Mixed (mainly consolidation)	241 (33.8%)	154 (30.3%)	87 (42.5%)	
Consolidation	22 (3.1%)	14 (2.8%)	8 (3.9%)	
None	15 (2.1%)	14 (2.8%)	1 (0.5%)	
**Shape of lesions**				
Nodular	6 (0.9%)	5 (1.0%)	1 (0.5%)	<0.001^*^
Linear	21 (2.9%)	20 (3.9%)	1 (0.5%)	
Patchy	467 (65.4%)	399 (78.4%)	68 (33.2%)	
Large patchy	205 (28.7%)	71 (14.0%)	134 (65.4%)	
None	15 (2.1%)	14 (2.8%)	1 (0.5%)	
**Halo sign**	115 (16.1%)	83 (16.3%)	28 (13.7%)	0.442^*^
Existence Period, day	11 (7–16.5)	11 (8–15)	10 (5–19.25)	0.663
**Reverse Halo Sign**	16 (2.2%)	11 (2.2%)	5 (2.4%)	0.785^*^
Existence Period, day	9 (5–16.5)	7 (4.5–14.5)	13 (8–16)	0.467
**Reticular patterns**	231 (32.4%)	108 (15.1%)	123 (60.0%)	**<0.001**
Existence Period, day	14 (9–20)	11 (8–15)	17 (12–23.5)	**<0.001**
**Air Bronchogram**	268 (37.5%)	155 (30.5%)	113 (55.1%)	**<0.001**
Existence Period, day	12 (8–18)	10 (7–15)	16 (11–22)	**0.006**
**Bronchiolectasis**	74 (10.4%)	24 (4.7%)	50 (24.4%)	**<0.001**
Existence Period, day	16.5 (11.25–23)	11 (6.75–11.25)	18 (14–25.75)	0.188
**Vascular enlargement**	284 (39.8%)	166 (32.6%)	118 (57.6%)	**<0.001**
Existence Period, day	12 (8–18)	11 (7–16)	15 (10–22.75)	<0.001^*^
**Crazy–paving sign**	113(15.8%)	95(18.7%)	18(8.8%)	**0.014**
Existence Period, day	11(7–14)	11(7–14)	11.5(7–14.25)	0.705
**Pleural thickening**	408 (57.1%)	242 (47.5%)	166 (81.0%)	**<0.001**
Existence Period, day	14 (9–20)	12 (8–17)	17 (11.25–23)	0.136
**Pleural traction**	325 (45.5%)	184 (36.2%)	141 (68.8%)	**<0.001**
Existence Period, day	15 (11–21)	14 (9–19)	18 (12–24)	**0.013**
**Pleural effusion**	40 (6.0%)	17 (3.3%)	23 (11.2%)	**<0.001**
Existence Period, day	18 (13.75–22.25)	15 (11–19)	18 (15.5–23)	**0.017**
**Mediastinal Lymphadenopathy**	34 (4.8%)	17 (3.3%)	19 (9.3%)	**0.002**
Existence Period, day	17.5 (11.75–23.25)	14 (10–19)	21 (15.5–25.5)	**0.011**
**Change of liver density**	8.41 ± 9.33	9.23 ± 8.19	7.05 ± 10.86	0.124

* *Wilcoxon's rank-sum test and Fisher's exact test were used when non-normal distribution or heterogeneous variance of the data*.

** *The periods were calculated from the first CT scan in 175 patients*.

*Existence period: The existence period of certain positive radiological findings*.

*The p value in bold indicates that P is less than 0.05 and is statistically significant*.

### Radiological Findings During Hospitalization

A total of 714 scans from 175 patients were evaluated including 509 scans from the non-severe group and 205 from the severe group. Detailed information on the first scan, last scan, and all scans are summarized in Supplementary Tables 2, 3, and Table 2, respectively. Patients underwent their first CT scan on day 7 (range: 5–10) of the course on average with no difference between the non-severe and severe groups.

During the period of **hospitalization**, patients in the severe group had a higher Extent Score than those in the non-severe group (11.16 ± 6.20 vs. 5.19 ± 3.54, *p* < 0.001, Table 2). The occurrence percentages of radiological signs, such as reticular patterns (*p* < 0.001), bronchiolectasis (*p* < 0.001), air bronchogram (*p* < 0.001), vascular enlargement (*p* < 0.001), pleural thickening (*p* < 0.001), pleural traction (*p* < 0.001), pleural effusion (*p* < 0.001), and mediastinal lymphadenopathy (*p* = 0.002), were significantly higher in the scans of severe group patients than in those of non-severe group patients. The crazy-paving sign was more commonly seen in the non-severe patients (*p* = 0.014).

**Figure 2:**
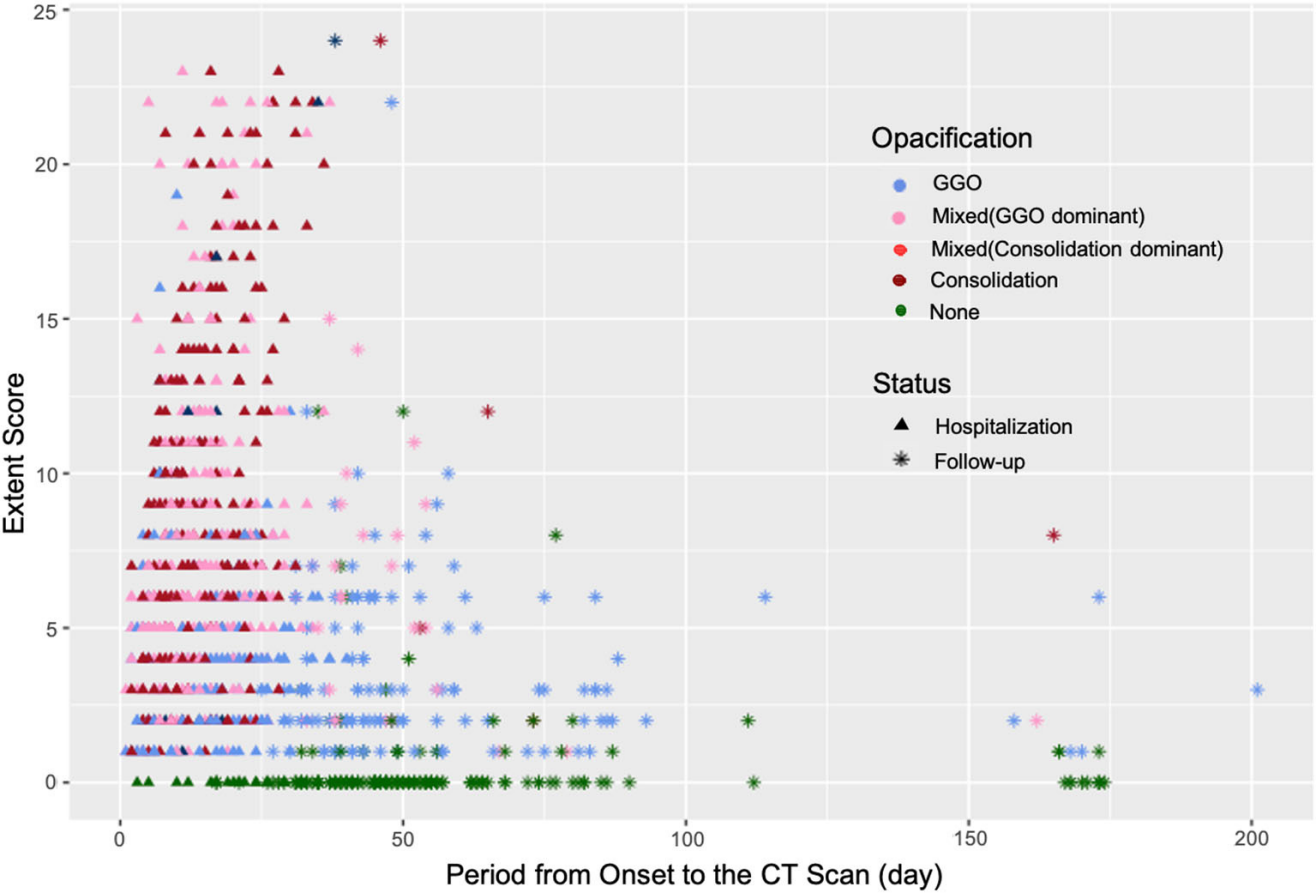
The changes in the Extent Score and opacification in the patients with COVID-19 during hospitalization and follow-ups. The opacities of lesions in the convalescent stage were mainly GGOs with a relatively high Extent Score.

The opacity was changed as the disease progressed. Overall, the evolution of the infiltrates from all the patients was observed from GGO to mixed consolidation and then from mixed consolidation to GGO at discharge (Figure 2). The normal CT findings before discharge were only observed in eight non-severe patients, accounting for 4.6% (8/175) of all participants.

### Radiological Findings at the Convalescence Stage

Among 175 participants, 135 (112 non-severe and 23 patients with severe COVID-19) underwent follow-up CT scans after discharge. Eight non-severe patients with complete pulmonary absorption at discharge and 32 patients (12 non-severe and 20 severe patients) who were lost to follow-ups were excluded. A total of 299 follow-up scans from 135 patients were studied. Lesions in 68 (50.4%) patients were completely absorbed with a median absorption period of 42.50 (IQR: 34.75–55.25) days. Patients with older age (46.81 ± 13.66 years old) and severe clinical type were more likely to have abnormal CT findings in the follow-up scans (Table 3). By the end of our observation, 67 patients still presented with pulmonary abnormalities. Among them, parenchymal bands were observed in 23 patients whereas opacifications were observed in the other 44 patients (Table 4).

**Table 3:** The comparison of clinical and CT manifestations during hospitalization between the patients with coronavirus disease 2019 (COVID-19) with normal and abnormal CT findings in the last follow-up scan after discharge.

	**All patients (***n*** = 135)**	**Normal (***n*** = 68)**	**Abnormal (***n*** = 67)**	***p*** **-value**
**Periods**				
Pulmonary Lesion Absorption Period, median (IQR), day	/	42.50 (34.75–55.25)	/	**/**
Period between discharge and the last follow-up CT scan, median (IQR), day	29.00 (28.00–54.50)	28.00 (27.75–32.25)	33.00 (28.00–62.50)	0.019^*^
Period between onset and the last follow-up CT scan, median (IQR), day	57.00 (47.50–76.50)	53.00 (47.00–65.25)	60.00 (48.50–87.00)	0.041^*^
**Clinical information**				
Age	44.15 ± 13.67	41.53 ± 13.26	46.81 ± 13.66	**0.024**
Gender, male/female	79/56	42/26	37/30	0.487
Underlying comorbidity yes/no	41/94	21/47	20/47	1.000
Clinical group: non-severe/severe	112/23	63/5	49/18	**0.003**
**CT manifestations from the scan at the peak stage ^†^**				
Morphology^†^				**<0.001**
Nodular	1 (0.7%)	1 (1.5%)	0 (0%)	
Large-path	42 (31.1%)	12 (17.6%)	30 (44.8%)	
Linear	8 (5.9%)	8 (11.8%)	0 (0%)	
Patchy	84 (62.2%)	47 (69.1%)	37 (55.2%)	
Extent^†^	7.34 ± 4.69	6.01 ± 3.73	8.69 ± 5.18	**0.001**
CT manifestations from the scan before discharge^*^				
Lesion involvement^*^				**0.001**
None	7 (5.2%)	6 (8.8%)	1 (1.5%)	
Diffuse	23 (17%)	5 (7.4%)	18 (26.9%)	
Multifocal	92 (68.1%)	47 (69.1%)	45 (67.2%)	
Unifocal	13 (9.6%)	10 (14.7%)	2 (4.5%)	
RUL^*^	86 (63.7%)	34 (50%)	52 (77.6%)	**0.001**
RML^*^	62 (45.9%)	21 (30.9%)	41 (61.2%)	**<0.001**
Distribution^*^				**0.001**
None	6 (4.4%)	5 (7.4%)	1 (1.5%)	
Both	41 (30.4%)	12 (17.6)	29 (43.3%)	
Subpleural	88 (65.2%)	51 (75%)	37 (55.2%)	
Morphology^*^				
None	7 (5.2%)	6 (8.8%)	1 (1.5%)	**0.002**
Nodular	1 (0.7%)	1 (1.5%)	0 (0%)	
Large-patchy	18 (13.3%)	3 (4.4%)	15 (22.4%)	
Linear	3 (2.2%)	3 (4.4%)	0 (0%)	
Patchy	106 (78.5%)	55 (80.9%)	51 (76.1%)	
Reticulation^*^	19 (14.1%)	2 (2.9%)	17 (25.4%)	**0.005**
Extent^*^	5.19 ± 4.17	3.68 ± 2.46	6.72 ± 4.95	**<0.001**

*Pulmonary lesion absorption period: The period from onset to the first CT scan with normal appearance*.

*In the comparison of CT findings between the two groups, only the imaging manifestations with significant difference were displayed in the table*.

† *CT manifestations from the scan at the peak stage*.

* *CT manifestations from the scan before discharge*.

*The p value in bold indicates that P is less than 0.05 and is statistically significant*.

**Table 4:** The clinical baseline and CT manifestations from the last scan in patients with COVID-19 with incomplete absorption status after discharge.

	**All (***n*** = 67)**	**Parenchymal band (***n*** = 23)**	**Opacification (***n*** = 44)**	***p*** **-value**
Age	46.81 ± 13.66	45.39 ± 14.16	47.40 ± 13.62	0.551
Gender, male/female	37/30	8/15	29/15	**0.030**
Underlying comorbidity yes/no	20/47	3/20	17/27	0.058
Clinical group: non-severe/severe	49/18	17/6	32/12	1.000
Period between onset and the last follow-up CT scan, median (IQR), day	60.00 (48.50–87.00)	75.00 (55.00–168.00)	58.00 (46.25–77.00)	0.0027^*^
**Pulmonary lobe involved**				
Right upper lobe	23 (34.3%)	3 (13.0%)	20 (46.5%)	**0.014**
Right middle lobe	23 (34.3%)	7 (30.4%)	16 (37.2%)	0.780
Right lower lobe	47 (70.2%)	13 (56.5%)	34 (79.1%)	**0.101**
Left upper lobe	25 (37.3%)	6 (26.1%)	19 (44.2%)	0.239
Left lower lobe	35 (52.2%)	13 (56.5%)	22 (51.2%)	0.875
**Location of lesions**				
Subpleural	50 (74.6%)	18 (78.3%)	32 (74.4%)	0.843
Both	17 (25.4%)	5 (21.7%)	12 (27.9%)	
**The existence of opacification**				**/**
GGO	/	/	37 (86.5%)	
Mixed (Mainly GGO)	/	/	4 (9.3%)	
Mixed (Mainly Consolidation)	/	/	2 (4.7%)	
Consolidation	/	/	1 (2.3%)	
Extent Score	/	/	2.66 ± 3.47	**/**
Halo Sign	1 (1.5%)	0 (0%)	1 (2.3%)	1.000^*^
Reverse Halo Sign	0 (0 %)	0 (0%)	0 (0%)	/
Reticular patterns	0 (0%)	0 (0%)	0 (0%)	/
Air Bronchogram	0 (0%)	0 (0 %)	0 (0%)	/
Bronchiolectasis	1 (1.5%)	0 (0%)	1 (2.3%)	1.000^*^
Vascular Enlargement	0 (0 %)	0 (0.00%)	0 (0%)	/
Crazy-paving sign	0 (0%)	0 (0%)	0 (0.00%)	/
Pleural thickening	1 (1.5%)	0 (0 %)	1 (2.3%)	1.000^*^
Pleural traction	0 (0%)	0 (0%)	0 (0%)	**/**
Pleural effusion	0 (0 %)	0 (0%)	0 (0%)	**/**
Mediastinal Lymphadenopathy	0 (0 %)	0 (0%)	0 (0%)	**/**

* *CT manifestations from the scan before discharge*.

*^†^CT manifestations from the scan at the peak stage*.

*The p value in bold indicates that P is less than 0.05 and is statistically significant*.

The parenchymal bands were usually distributed in the subpleural regions and GGO was the main manifestation of opacification. After comparison, patients with incomplete lesion absorption were detected to have diffuse lesions with a higher Extent Score both at the peak stage and before discharge (Table 3).

**Figure 3:**
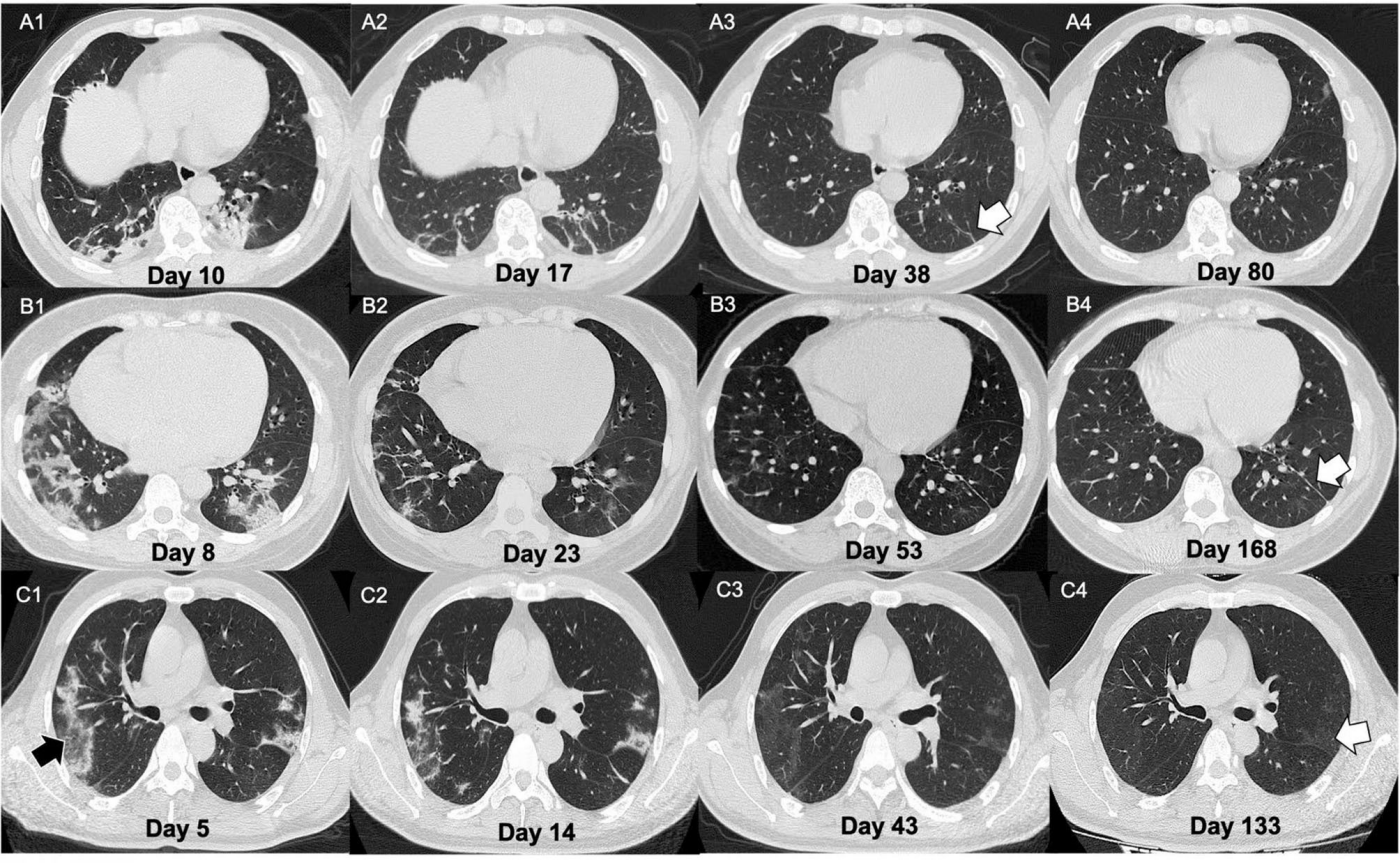
Illustration of three patients with different CT manifestations in the follow-ups. **(A)** A 51-year-old male presented with fever for 9 days. On admission, the chest CT showed multiple patchy lesions (mixed lesions with dominant consolidation) and fibrotic changes **(A1)**. The man was then confirmed as infected with COVID-19 by nucleic acid amplification test NAAT. After treatment, the lesions showed evident absorption on day 17 from onset **(A2)**. He was discharged on day 25 of the course. Some parenchymal bands (white arrow) remained in week 2 of follow-up **(A3)** but were absolutely absorbed on day 80 after onset **(A4)**. **(B)** A 34-year-old female presented with fever and diarrhea for 3 days. On admission, the chest CT showed large patchy lesions (mixed lesions with dominant GGO) **(B1)**. The woman was then confirmed as COVID-19 by NAAT. After treatment, the lesions showed evident absorption. On day 23 of the course **(B2)**, the patient was discharged. Parenchymal bands could be observed at 1 and 4 months after discharge [**(B3,B4)**, white arrow]. **(C)** A 48-year-old male presented with fever and cough for 24 days. On admission, his chest CT showed multiple patchy lesions with consolidative changes **(C1)** with reversed halo signs observed (black arrow). The man was then confirmed as COVID-19 by NAAT. After treatment, the lesions showed evident absorption on day 33 from onset **(C2)**. The patient was discharged 2 days later. However, pulmonary lesions with GGO opacification were not completely absorbed at the 1- and 3-month follow-ups [**(C3,C4)**, white arrow].

It was notable that the parenchymal bands in the nine patients (9/23, 39.1%) disappeared in the last follow-up scan. The typical cases are demonstrated in Figure 3. We have compared the clinical, CT manifestations among the 23 patients with residual parenchymal bands and those nine patients mentioned above. Patients with unabsorbed fibrosis-like lesions had a higher percentage of lesion involvement in the right upper lobe with a patchy/large-patchy appearance at discharge (Table 5).

**Table 5:** The comparison of clinical manifestations between the patients with COVID-19 with absorbed parenchymal bands and residual parenchymal bands in the last follow-up scan after discharge.

	**Patients with absorbed parenchymal bands (***n*** = 9)**	**Patients with residual parenchymal bands (***n*** = 23)**	***p*** **-value**
**Periods**			
Pulmonary Lesion Absorption Period, median (IQR), day	40.00 (30.25–41.50)	/	/
Period between discharge and the latest follow-up CT scan, median (IQR), day	28.00 (27.00–29.00)	55.00 (29.00–138.50)	0.034^*^
Period between onset and the latest follow-up CT scan, median (IQR), day	40.00 (30.25–41.50)	75.00 (55.00–168.00)	0.068^*^
**Clinical information**			
Age	45.39 ± 14.16	40.11 ± 14.04	0.304
Gender, male/female	42/26	37/30	0.132
Underlying comorbidity yes/no	21/47	20/47	0.604
Clinical group: non-severe/severe	63/5	49/18	1.000
**Morphology ^†^**			
Nodular	0 (0%)	0 (0%)	**<0.002**
Large-patchy	3 (33.3%)	12 (52.2%)	
Linear	4 (44.4%)	0 (0%)	
Patchy	2 (22.2%)	11 (47.8%)	
RUL^†^	2 (22.2%)	18 (78.3%)	**0.005**
Morphology^*^			
None	2 (22.2%)	1 (4.3%)	**0.004**
Nodular	0 (0%)	0 (0%)	
Large-patchy	1 (11.1%)	3 (13%)	
Linear	3 (33.3%)	0 (0%)	
Patchy	3 (33.3%)	19 (83.6%)	

*The p value in bold indicates that P is less than 0.05 and is statistically significant*.

† *CT manifestations from the scan at the peak stage*.

* *CT manifestations from the scan before discharge*.

**Figure 4:**
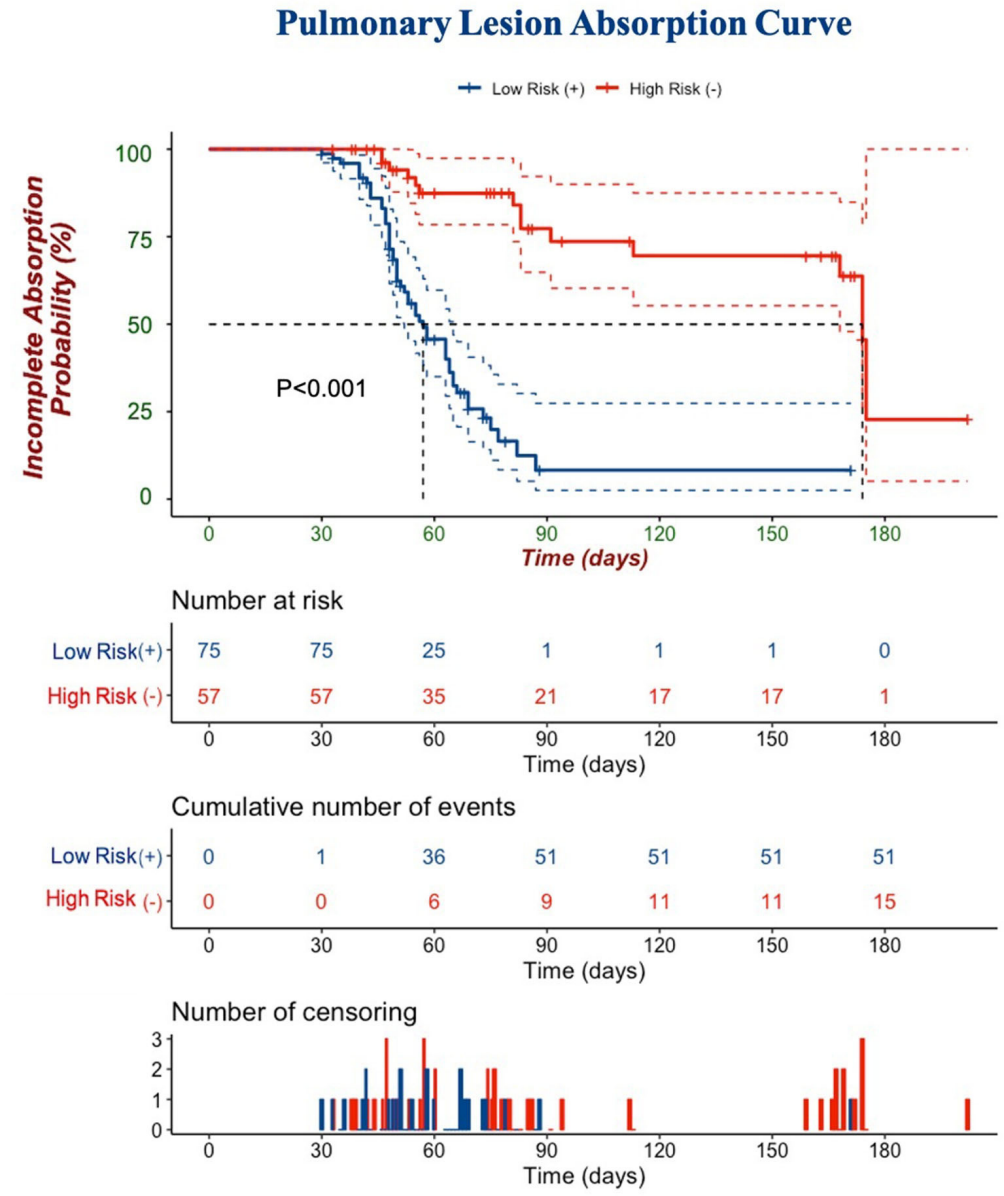
A Kaplan–Meier curve is depicted to demonstrate the ability of Cox regression to discriminate complete and incomplete pulmonary absorption in patients. Cumulative number of events: cumulative number of patients who had pulmonary lesions completely absorbed. The number of censorings: the number of patients who had pulmonary lesions completely absorbed or lost follow-ups before the end of our observation.

In the univariate Cox regression analyses, one clinical, two laboratories, and five CT features from the last scan before discharge were significantly associated with incomplete absorption of pulmonary lesions (all *p* < 0.05). The Cox proportional hazards model retained age [hazard ratio (*HR*), 0.95; 95% *CI*, 0.95–0.99; *p* = 0.003], LDH (*HR*, 0.99; 95% *CI*, 0.99–1.00; *p* = 0.026), procalcitonin (*HR*, 8.72; 95% *CI*, 1.04–73.03; *p* =0.046), diffuse distribution (*HR*, 0.28; 95% *CI*, 0.09–0.92; *p* =0.013), subpleural abnormalities (*HR*, 2.15; 95% *CI*, 1.17–3.92; *p* =0.013), linear lesions (*HR*, 4.58; 95% *CI*, 1.22–17.11; *p* =0.002), nodular lesions (*HR*, 33.07; 95% *CI*, 3.58–305.74; *p* =0.002), and the existence of pleural traction (*HR*, 0.41; 95% *CI*, 0.22–0.78; *p* = 0.006) as independent predictive factors (Table 6). The best cut-off value of this Cox model was −0.85 with a C-index of 0.747 ± 0.033 that could stratify patients with complete and incomplete pulmonary absorption successfully (*p* < 0.001, log-rank test). By Kaplan–Meier analysis, the probability of patients of the low-risk group to have pulmonary lesions fully absorbed within 90 days reached 91.7%, whereas the probability in the high-risk group was only 22.7% (Figure 4).

**Table 6:** Results of univariate and multivariate Cox proportional hazard regression analyses.

**Variable**		**Univariate Cox hazard analysis**		**Multivariate Cox hazard analysis**	
		**HR (95% CI)**	***p*** **-value**	**HR (95% CI)**	***p*** **-value**
Clinical Information (3)	Age	0.97 (0.95–0.99)	<0.001	0.95 (0.95–0.99)	0.003
	LDH	0.99 (0.99–1)	<0.001	0.99 (0.99–1.00)	0.026
	Procalcitonin^b^	1.9 (1.1–3.1)	0.015	8.72 (1.04–73.03)	0.046
CT manifestations before discharge^a^ (5)	Lesion involvement-diffuse^a^, ^b^	0.22 (0.087–0.55)	<0.001	0.28 (0.09–0.92)	0.036
	Distribution-subpleural^a^, ^b^	1.8 (1.1–3.2)	0.030	2.15 (1.17–3.92)	0.013
	Morphology-liner^a^, ^b^	9.0 (2.7–30)	<0.001	4.58 (1.22–17.11)	0.024
	Morphology -Nodular^a^, ^b^	31 (3.6–270)	0.002	33.07 (3.58–305.74)	0.002
	Pleural traction^a^	0.4 (0.23–0.7)	0.001	0.41 (0.22–0.78)	0.006

a *The CT findings were analyzed based on the last chest CT scan before discharge*.

b *The “total absorption” was set as the endpoint of the Cox Regression model, therefore, features with HR value over 1.0 were regarded as the protective factors*.

## Discussion

After the worldwide outbreak of SARS, MERS, and COVID-19, coronaviruses have attracted great attention in the global healthcare system (20–24). Chest CT scans have been widely used as screening methods to identify patients with COVID-19 pneumonia. Despite the debate around the diagnostic importance of CT and NAAT, a chest CT is currently no longer recommended as the first choice of COVID-19 diagnosis due to the radiation risk. However, it is valuable to study the dynamic changes of COVID-19 on the multiple chest CT scans, which could help us optimize the management and follow-up plans of the disease (7, 25).

A total of 714 serial scans from 175 patients during hospitalization and 299 follow-up scans from 135 patients obtained in our cohort provided us with an opportunity to study the longitudinal pulmonary changes in COVID-19. To the best of our knowledge, although many studies have been conducted to describe the longitudinal pulmonary changes in the patients with COVID-19 in the acute period, few have investigated the absorption of lesions at the convalescent stage. Lesions of COVID-19 induced pneumonia were distributed mainly in the subpleural region and progressed from patches to large patches with hardly noticeable pleural changes. With the progression of the disease, density changes were seen with a regular pattern of GGO-consolidation-GGO, which was aligned with previous reports (26–28). The pathological findings revealed that edema, proteinaceous exudate, and hyperplasia of pneumocytes with patchy inflammatory cellular infiltration and the absence of hyaline membranes were histological changes in the early stage of COVID-19 which presented as GGOs at the onset (26, 29). Diffuse alveolar damage with cellular fibro-myxoid exudates corresponded to the acute respiratory distress syndrome (ARDS), presented with a consolidation-predominant appearance on the CT images (5). In the convalescent period, GGOs might also represent the existence of proteinaceous or fibro-myxoid exudates that would be absorbed gradually. Since it is difficult to distinguish the GGO lesions in different stages, it is recommended to take the time course into consideration when making diagnoses and differential diagnoses, rather than merely basing them on the CT findings. With the progression of density, the areas of involved pulmonary tissues, which was reflected by the Extent Score, decreased significantly as well (at peak vs. at discharge: 7.34 ± 4.69 vs. 5.19 ± 4.17, *p* < 0.001). Several studies have addressed the importance of the Extent Score in the evaluation of the severity of patients with COVID-19 (17, 30, 31). Moreover, according to this study results, the opacities changed before shrinkage of the affected areas could be observed. Therefore, to evaluate the severity of the disease, we thought attention should be given to the opacification pattern of lesions as well.

Based on our findings, several radiological features possibly indicated the severity of the disease at the acute stage. It is worth mentioning that the CT sign of vascular enlargement was frequently seen in severe patients during hospitalization (57.6%), but was rarely seen in non-severe ones or patients after discharge. It was reported that in patients with COVID-19, due to inflammation and the activation of arteriovenous anastomoses, venous blood flow increases and results in the vascular dilatation in the venous compartment (32, 33). This could help to understand the sign of vascular enlargement in severe patients with COVID-19 (34). Since the plain CT scans in this study limited the accurate recognition of blood vessels, we suggest that further studies with enhanced chest CT data be conducted to explore the reason for vascular enlargement in patients with COVID-19.

According to the guidelines provided by the Government of China, all COVID-19 patients should undergo at least two follow-up chest CT scans at 2 weeks and 1 month, respectively, after discharge. For those patients with unabsorbed lesions detected after discharge, it was recommended to take the CT scans several months later until complete absorption was observed. However, recommendations were raised based on our experiences with SARS. In this study, the follow-up period of our 135 patients ranged from 8 to 174 days after discharge. In SARS-related studies, 17 patients were followed up, consolidation generally resolved completely (*n* = 4) or to minimal residual opacities; six (55%) of 11 patients with ground-glass opacities had substantial residual disease (CT scores >5) on final scans (35). Compared with the residual pulmonary consequences in SARS patients, the percentage and extensiveness of these pulmonary abnormalities were increasingly lower (36, 37). In the current study, 68 patients had their lesions totally absorbed, with a median duration of 42.50 days since onset. Among the 67 patients with unabsorbed abnormalities, approximately one-third presented with parenchymal bands, while opacities were observed in the other 40 patients with GGOs as the predominant presentation (38, 39). The patients with residual opacities were mostly male with a shorter follow-up duration which indicated that the parenchymal band might be the next stage after GGO absorption.

The parenchymal band is widely accepted as a consequence of linear atelectasis distal to the narrowed bronchus, or pleuro-parenchymal fibrosis, which could potentially affect the pulmonary function. This fibrosis-like abnormality was reported by Yu et al. and Gregory Antonio et al. as one of the consequences of coronavirus infection with a limited follow-up duration (14, 40). In this study, the parenchymal bands were absorbed in nine patients (9/68, 13.2%) with a median absorption period of 40 days since the onset (range: 26–174 days). After comparison between patients with absorbed and unabsorbed parenchymal bands, only the location of the pulmonary lesions (especially in the right upper lobe) demonstrated a difference. Based on these findings, we assumed that the absorbed parenchymal bands may only represent temporary atelectasis and that most of them would be absorbed gradually. As the absorption duration of the parenchymal bands varies significantly, it is recommended for patients with fibrosis-like residuals to have long-term follow-ups.

At the beginning of the outbreak, according to guidelines from the government of China, patients with COVID-19 in this study were not required to undergo a pulmonary function test. A few studies have mentioned that the average pulmonary function was normal in patients after mild/moderate COVID-19. The patients after severe/critical COVID-19 generally had lower lung volumes that were still within the normal range (41, 42).

Since nearly half of the patients presented with residual pulmonary lesions in their last follow-up scan, it was important to determine which factors could actually affect the absorption status of the COVID-19 related pulmonary lesions. A set of eight clinical and CT features were selected as risk factors. Among them, high procalcitonin level, subpleural distribution of lesions, and lesions with liner and nodular shape in CT images before discharge worked as protective factors since these features indicated limited lesion involvement at the time of discharge, which might be due to an adaptive, beneficial, and therapeutic response to the pathogens. Procalcitonin has been widely used in identifying infectious diseases in recent years and is now recognized as a validated serological marker for the identification of microbial infections. This is of great significance in the diagnosis of early infection and the assessment of the extent of early infection (43). We assumed that the early detection of a high level of procalcitonin might help the patients that receive early intervention treatment, thus resulting in the faster absorption of the lesions. The peripheral subpleural pulmonary lobules are well-developed with rich blood flow and lymph system, facilitating the absorption of infiltrates, which could explain why subpleural distribution of lesions worked as a protective factor. A high level of LDH, older age, the existence of diffuse lesion distribution, and pleural traction served as risk factors to delay absorption that was consistent with the previous studies. Among them, LDH, as one of the most important enzymes for anaerobic glycolysis and gluconeogenesis, was frequently elevated in severe patients, and pleural traction, a sign of pleural adhesion and hypertrophy caused by fibrin deposition, is also a sign of severe and long-term inflammation (44, 45). The current study results suggest that those patients were more likely to have residual lesions in the lungs. We could consider that in a young patient with low LDH and procalcitonin levels and subpleural linear/nodular pulmonary lesions before discharge, it may not be necessary to perform follow-up CT scans.

Based on these predictive factors, our Cox model could efficiently stratify patients into two groups: almost all patients in the low-risk group had their lesions absorbed in 3 months, while 50% of the patients in the high-risk group had pulmonary residuals even after 6 months. To monitor the absorption status and minimize radiation damage, we suggest that 3 months since onset could be an optimal timepoint to monitor the absorption status in discharged patients with COVID-19 rather than a 1-month CT follow-up.

As far as we know, this is the first study to report detailed time-course-based radiological findings from onset to 1-year follow-up. There are still several limitations in this study. First, we did not collect the dynamic changes in laboratory tests and lacked pulmonary function results. Second, the treatment might vary from February until now, which might bring changes to the radiological course of COVID-19. Third, we only enrolled the adult patients for this study; therefore, our results could not be generalized to children or pregnant women.

In conclusion, we found that the development of COVID-19 lesions evolved from GGO to consolidation and then from consolidation to GGO. The CT manifestations from scans at the peak stage and before discharge could help to predict the absorption status of pulmonary lesions. The parenchymal bands were observed to be absorbed in some COVID-19 cases and thus should not be regarded as irreversible fibrosis. Our Cox regression analysis indicated that 3 months since onset could be an optimal timepoint for radiological follow-up in the discharged patients.

## Data Availability Statement

The datasets presented in this article are not readily available because approval from the relevant authorities is required. Requests to access the datasets should be directed to susan_lyp@163.com.

## Ethics Statement

The studies involving human participants were reviewed and approved by Ethics Committee of Huashan Hospital Affiliated to Fudan University. Written informed consent for participation was not required for this study in accordance with the national legislation and the institutional requirements.

## Author Contributions

YL, LL, and BY conceived and designed this study. XL, YZhe, AX, XQ, and XY conducted the study and collected important background data. YZha, DW, and NM drafted the manuscript. All authors read and approved the final manuscript.

## Funding

This project was supported by the Clinical Research Plan of SHDC (Grant No. SHDC2020CR4069), the Youth Program of National Natural Science Foundation of China (Fund No. 81901697), the Shanghai Sailing Program (Grant No. 21YF1404800), the Shanghai Municipal Science and Technology Major Project (No. 2018SHZDZX01), and the ZJ Lab. All the funding sources played no roles in the study.

## Conflict of Interest

The authors declare that the research was conducted in the absence of any commercial or financial relationships that could be construed as a potential conflict of interest.

## Publisher's Note

All claims expressed in this article are solely those of the authors and do not necessarily represent those of their affiliated organizations, or those of the publisher, the editors and the reviewers. Any product that may be evaluated in this article, or claim that may be made by its manufacturer, is not guaranteed or endorsed by the publisher.
