# Supplementary material for: Longitudinal Radiological Findings in Patients With COVID-19 With Different Severities: From Onset to Long-Term Follow-Up After Discharge
Source: Front Med (Lausanne). 2021 Sep 21;8:711435. doi: 10.3389/fmed.2021.711435 (PMC8490620; doi:10.3389/fmed.2021.711435)
Supplement: Supplementary file 1 [file Data_Sheet_1.docx]

**A1. Criteria for clinical type of COVID-19 (the Diagnosis and Treatment guide for COVID-19, 8th Trail Edition and WHO guideline)**

| China Classification | WHO Classification | Standard |
| --- | --- | --- |
| Mild type | **Mild type**  (2 clinical syndromes including mild-illness and pneumonia) | Patients with uncomplicated upper respiratory tract viral infection, may have non-specific symptoms such as fever, fatigue, cough (with or without sputum production), anorexia, malaise, muscle pain, sore throat, dyspnea, nasal congestion, or headache. Rarely, patients may also present with diarrhea, nausea and vomiting; no signs of pneumonia on images;^1&2^ |
| Moderate type |  | Fever and respiratory tract symptoms; imaging findings of **pneumonia**; ^1&2^ |
| Severe type | **Severe type** (2 clinical syndromes including severe pneumonia and ARDS) | Meet any of the following conditions: 1) shortness of breath, RR ≥ 30 times/min; 2) SPO_2_ ≤ 93% at rest; 3) PaO_2_/ FiO_2_ ≤ 300 mmHg (1 mmHg = 0.133 kPa). ^1&2^  **ARDS**: When PaO2 is not available, SpO2/FiO2 ≤ 315 suggests ARDS (including in non-ventilated patients). ^2^ |
| Critical type | **Critical type** (2 clinical syndromes including sepsis and septic shock) | Meet any of the following conditions: 1) respiratory failure with the requirement of mechanical ventilation; 2) shock; 3) failure of other organs with the requirement of ICU monitoring and treatment.^1^  Signs of **organ dysfunction** include: altered mental status, difficult or fast breathing, low oxygen saturation, reduced urine output, fast heart rate, weak pulse, cold extremities or low blood pressure, skin mottling, or laboratory evidence of coagulopathy, thrombocytopenia, acidosis, high lactate or hyperbilirubinemia.^2^  **Septic Shock**: persisting hypotension despite volume resuscitation, requiring vasopressors to maintain MAP ≥ 65 mmHg and serum lactate level > 2 mmol/L.^2^ |

RR: respiratory rate; SPO_2_: oxygen saturation; PaO_2_: partial pressure of oxygen; FiO_2_: fraction of inspired oxygen; ICU: intensive care unit; ARDS: Acute Respiratory Distress Syndrome

1: The standard of the clinical type in China Classification;

2: The standard of the clinical type in WHO Classification.

**A2. Imaging Manifestations in the first CT scan**

| Imaging manifestation | All Scans  (n = 175) | Non-Severe Group Scans (n=132) | Severe Group Scans (n=43) | *p* value |
| --- | --- | --- | --- | --- |
| Period between Onset and the 1^st^ CT Scan, median (1/4-3/4 quantile), day | 7 (5-10) | 7 (4-10) | 7 (4-11.5) | 0.581 |
| Involved Lobes: |  |  |  |  |
| Right Upper Lobe | 128 (73.1%) | 90 (68.2%) | 38 (88.4%) | **0.017** |
| Right Middle Lobe | 105 (60.0%) | 75 (56.8%) | 30 (69.8%) | 0.185 |
| Right Lower Lobe | 142 (81.1%) | 105 (79.5%) | 37(86.1%) | 0.470 |
| Left Upper Lobe | 124 (70.9%) | 88 (66.7%) | 36 (83.7%) | 0.052 |
| Right Lower Lobe | 151 (86.3%) | 112 (84.%) | 39 (90.7%) | 0.476 |
| Location of Lesions: |  |  |  |  |
| Subpleural | 110 (62.9%) | 91 (68.9%) | 19 (44.2%) | **0.019*** |
| Central | 5 (2.9%) | 3 (2.3%) | 2 (4.7%) |  |
| Both | 59 (33.7%) | 38 (28.8%) | 21 (48.8%) |  |
| None | 1 (0.6%) | 0 (0%) | 1 (2.3%) |  |
| Extent of Lesions: |  |  |  |  |
| Unifocal | 11 (6.3%) | 10 (7.6%) | 1 (2.3%) | **0.002*** |
| Multi-focal | 115 (65.7%) | 94 (71.2%) | 21 (48.8%) |  |
| Diffuse | 48 (27.4%) | 28 (21.2%) | 20 (16.5%) |  |
| None | 1 (0.6%) | 0 (0%) | 1 (2.3%) |  |
| Extent Score | 6.61±4.76 | 5.61±3.37 | 9.65±6.76 | **<0.001*** |
| The Existence of Opacification |  |  |  |  |
| GGO | 35 (20.0%) | 26 (19.7%) | 9 (20.9%) | 0.161* |
| Mixed (Mainly GGO) | 80 (45.7%) | 57 (43.2%) | 23 (53.5%) |  |
| Mixed (Mainly Consolidation) | 52 (29.7%) | 44 (33.3%) | 8 (18.6%) |  |
| Consolidation | 7 (4.0%) | 5 (3.8%) | 2 (4.7%) |  |
| None | 1 (0.6%) | 0 (0%) | 1 (2.3%) |  |
| Shape of Lesions: |  |  |  |  |
| Nodular | 8 (4.6%) | 8 (6.1%) | 0 (0%) | **<0.001*** |
| Linear | 2 (1.1%) | 2 (1.5%) | 0 (0%) |  |
| Patchy | 126 (72.0%) | 106 (80.3%) | 20 (46.5%) |  |
| Large patchy | 38 (21.7%) | 16 (12.1%) | 22 (51.2%) |  |
| None | 1 (0.6%) | 0 (0%) | 1 (2.3%) |  |
| Halo Sign | 42 (24.0%) | 31 (23.5%) | 11 (25.6%) | 0.365* |
| Reverse Halo Sign | 7 (4.0%) | 4 (3.0%) | 3 (7.0%) | 0.785* |
| Reticulation | 52 (29.7%) | 30 (22.7%) | 22 (51.2%) | **0.008** |
| Air Bronchogram | 74 (42.3%) | 51 (38.6%) | 23 (53.5%) | 0.125 |
| Bronchiectasis | 20 (11.4%) | 11 (8.3%) | 9 (20.9%) | **0.049** |
| Vascular Enlargement | 85 (48.6%) | 59 (44.7%) | 26 (60.5%) | 0.105 |
| Crazy-paving sign | 35(20.0%) | 30(27.3%) | 5(11.6%) | 0.114 |
| Pleural thickening | 102 (58.3%) | 72 (54.6%) | 30 (69.8%) | 0.114 |
| Pleural traction | 62 (35.4%) | 42 (31.8%) | 20 (46.5%) | 0.117 |
| Pleural effusion | 7 (4.0%) | 4 (3.0%) | 3 (7.0%) | 0.365* |
| Mediastinal Lymphadenopathy | 7 (4.0%) | 3 (2.3%) | 4 (9.3%) | 0.063* |

**A3. Imaging Manifestations in the last CT scan before discharge**

| Imaging manifestation | All Scans  (n = 175) | Non-Severe Group Scans (n=132) | Severe Group Scans (n=43) | *p* value |
| --- | --- | --- | --- | --- |
| Period between Onset and the last CT Scan, median (1/4-3/4 quantile), day | 22 (16.5-26) | 20 (16-25) | 25 (20.5-29) | **0.004** |
| Scan Earlier/Not earlier than the 2nd Negative NAAT | 100/75 | 73/59 | 27/16 | 0.494 |
| Non-lesion Scan Earlier/Not earlier than the 2nd Negative NAAT | 6/2 | 6/2 | 0/0 | 1.000* |
| Involved Lobes: |  |  |  |  |
| Right Upper Lobe | 120 (68.6%) | 79 (59.9%) | 41 (95.4%) | **<0.001** |
| Right Middle Lobe | 94 (53.7%) | 57 (43.2%) | 37 (86.1%) | **<0.001** |
| Right Lower Lobe | 144 (82.3%) | 102 (77.3%) | 42 (97.7%) | **0.005** |
| Left Upper Lobe | 119 (68.0%) | 78 (59.2%) | 41 (95.4%) | **<0.001** |
| Right Lower Lobe | 150 (85.7%) | 107 (81.1%) | 43 (100.0%) | **0.005** |
| Location of Lesions: |  |  |  |  |
| Subpleural | 102 (58.3%) | 87 (65.9%) | 15 (34.9%) | **<0.001*** |
| Central | 0 (0%) | 0 (0%) | 0 (0.0%) |  |
| Both | 65 (37.1%) | 37 (28.0%) | 28 (65.1%) |  |
| None | 8 (4.6%) | 8 (6.1%) | 0 (0%) |  |
| Extent of Lesions: |  |  |  |  |
| Unifocal | 13 (7.4%) | 13 (9.9%) | 0 (0%) | **<0.001*** |
| Multi-focal | 106 (60.6%) | 94 (71.2%) | 12 (27.9%) |  |
| Diffuse | 48 (27.4%) | 17 (12.9%) | 31 (72.1%) |  |
| None | 8 (4.6%) | 8 (6.1%) | 0 (0%) |  |
| Extent Score | 6.61±4.76 | 4.64±3.16 | 12.07±6.27 | **<0.001*** |
| The Existence of Opacification |  |  |  |  |
| GGO | 52 (29.7%) | 46 (34.9%) | 6 (14.0%) | **<0.001*** |
| Mixed (Mainly GGO) | 67 (37.1%) | 51 (38.6%) | 16 (37.2%) |  |
| Mixed (Mainly Consolidation) | 44 (25.1%) | 24 (18.2%) | 20 (46.5%) |  |
| Consolidation | 4 (2.3%) | 3 (2.3%) | 1 (2.3%) |  |
| None | 8 (4.6%) | 8 (6.1%) | 0 (0%) |  |
| Shape of Lesions: |  |  |  |  |
| Nodular | 1 (0.6%) | 1 (0.8%) | 0 (0%) | **<0.001*** |
| Linear | 3 (1.7%) | 3 (2.3%) | 0 (0%) |  |
| Patchy | 129 (73.7%) | 112 (84.9%) | 17 (39.5%) |  |
| Large patchy | 34 (19.4%) | 8 (6.1%) | 26 (60.5%) |  |
| None | 8 (4.6%) | 8 (6.1%) | 0 (0%) |  |
| Halo Sign | 15 (8.6%) | 12 (9.1%) | 3 (7.0%) | 1.000* |
| Reverse Halo Sign | 2 (1.1%) | 2 (1.5%) | 0 (0%) | 1.000* |
| Reticulation | 41 (23.4%) | 16 (12.1%) | 25 (58.1%) | **<0.001** |
| Air Bronchogram | 31 (17.7%) | 16 (12.1%) | 15 (34.9%) | **0.002** |
| Bronchiectasis | 10 (5.7%) | 1 (0.8%) | 9 (20.9%) | **<0.001*** |
| Vascular Enlargement | 44 (25.1%) | 23 (17.4%) | 21 (48.8%) | **<0.001*** |
| Crazy-paving sign | 0 (0%) | 0 (0%) | 0 (0%) | **/** |
| Pleural thickening | 84 (48.0%) | 51 (38.6%) | 33 (76.7%) | **<0.001*** |
| Pleural traction | 68 (38.9%) | 38 (28.8%) | 30 (69.7%) | **<0.001*** |
| Pleural effusion | 11 (0.6%) | 3 (2.2%) | 8 (18.6%) | **<0.001*** |
| Mediastinal Lymphadenopathy | 4 (2.3%) | 1 (0.8%) | 3 (7.0%) | **0.046*** |
